# Cases of Cyanosis, Attended with, and Probably Dependent upon, Collapse of the Lung

**Published:** 1863-12

**Authors:** John Bartlett

**Affiliations:** Chicago, Ill.


ARTICLE XXXIV.

### CASES OF CYANOSIS, ATTENDED WITH, AND PROBABLY DEPENDENT UPON, COLLAPSE OF THE LUNG.

By JOHN BARTLETT, M.D., of Chicago, Ill.

Read before the Chicago Medical Society, September 11, 1863.

Case I. In July, 1858, Mrs. S. W. gave birth to a healthy child. On the eighth day it sickened. The mother attributed the symptoms to the bad quality of her milk. Before she bore a child, a tumor, said by her physicians to be hard cancer, had been removed from the breast. All of her other children, seven in number, had suffered as this one, and at the same age. In three cases, the disease had proved fatal.

The child was much emaciated. The general surface had been noticed occasionally to be of a dark red color, and the face of a purple hue. There was a severe diarrhœa. But the most prominent symptom was long-continued paroxysms of intense pain. Three convulsions were said to have occurred within a few hours past. Anodynes and astringents were given; a suitable wet-nurse was obtained; liniments were applied to the chest; and the child was laid upon the right side. Thirty-six hours later, its diarrhœa continued, its tendency to convulsions was greater, and the cyanosis more decided. Occasionally the respiration was much hurried and labored; the respiratory movements, at one time, reaching eighty per minute. No very significant auscultatory phenomena were observed. A slight ronchus, and diminished respiratory murmur were constantly noticed on the left side. These signs were much more marked on some days than on others. The periods of greatest diminution of the murmur were not coincident with those of the greatest acceleration of breathing: on the contrary, puerile respiration was always distinct, whenever the breathing was rapid.

Probably, the exaggeration of the vesicular murmur in the healthy portion of the lung rendered inaudible the circumscribed diminished sound. It was during the hours of greatest feebleness that the greatest embarrassment of respiration occurred. Some days of improvement followed the treatment; but all the symptoms returning, in an aggravated form, after ten days of illness, death resulted.

An autopsy, eighteen hours after death, revealed all the abdominal organs somewhat congested with dark blood; the intestines were congested in all their coats, but there was no evidence of inflammation.

Posteriorly, in each lobe of both lungs, but particularly in the middle lobe of the right lung, and in the lower lobe of both lungs, there were lobules in a state of carnification. This condition was mostly superficial; but in spots, it extended into the lung the width of the finger. Probably one-tenth of the substance of the organs was involved. Upon moderate inflation, the most of the affected lobules resumed their normal state, the greater number collapsing when the inflation ceased. Some could not be expanded.

The cavities and vessels of the heart were natural. The valve of the foramen ovale* was imperfect, presenting an opening of one-tenth of an inch in diameter. The ductus arteriosus was patent to the like extent.

CASE II. On the 26th of April, 1863, Mrs. J. D., a strong, healthy woman, aged about forty years, was attacked with violent spasms. She was, as was supposed, in the eighth month of pregnancy. As the convulsions occurred in lieu of an antici-

* This statement as to the deficiency of the valve is taken from notes written at the time of observation. The experience detailed below is sufficient to cause me to set aside that opinion. Probably, more careful study of the anatomy would have shown the valve perfect. I am the more disposed to this conclusion since Dr. J. R. ALLEN, then Professor of Obstetrics in the Iowa University, who witnessed the examination, expressed doubts as to the abnormal state of the parts in question.

In the examination of the heart, in the following case, I determined to make certain of its condition, especially in regard to the foramen ovale. The septum auricularum was viewed in many directions. The heart being held in the hand in a natural position, a current of water was directed against the left side of the septum, floating up the valve. The result of this unusually careful examination was the conviction that the valve was deficient in its anterior and inferior third: it was so recorded.

pated chill, and as no other cause for them, other than malarial influence, was discovered, antiperiodics were trusted to for relief. The result was entirely satisfactory.

On the 18th of May, after an easy labor, she was the mother of a small, feeble child. It did not breathe at once; but respiration was soon apparently well established. The infant was placed in a sheet and allowed to remain there half an hour. An unusual amount of phlegm was early noticed to pass from the air passages; it was found upon the pillow, or in the mouth of the child; occasionally a cough would extrude it. For the first few days, it was incapable of swallowing; but there was no serious difficulty in respiration, except when an attempt to excite deglutition by passing fluids into the fauces, suspended breathing for some moments, and cyanosed the child. These attacks of suspended respiration, after the first five days of life, came on independently of extraneous causes.

As the child lay quiet, the face would gradually become livid, the breathing apparently ceasing, and death seeming imminent. From careful study, it was established that this was a real choking, precisely similar to that produced by allowing fluids to pass into the throat. The exciting cause was the thick, tenacious phlegm above alluded to. When there was strength to cough this off, the paroxysms were arrested. When the swab removed it, animation was restored. In no instance, during these attacks, did the efforts of the nurse to dislodge this mucus entirely fail. The trachea contained it in abundance. Tubes passed into this passage came out covered with it. After two weeks, the violence and frequency of the paroxysms diminished.

Desiring to demonstrate to the members of the Society before which the paper was to be read the actual patency of the foramen, this arrangement was effected. The septum was laid, the left side downward, loosely over the end of a perforated cork, and secured in place by pins, at the margin. A gentle stream of water was then passed through the cork, from below upward; and, to the astonishment of all who had previously examined the heart, the valve sprang to its place, completely closing the foramen.

Conviction that this is not the first instance in which an imperfect examination of the valves of the heart has led to error, is my apology for giving the history of this mistake in detail.

After five weeks, they entirely ceased for a fortnight; but again recurring, the child sank, after seven weeks more of suffering. In the last hours of life, the respiration was hurried, and there were slight convulsive movements of the muscles of the eye.

During the three months of its existence, this child scarcely swallowed enough to sustain a vigorous infant one week; and yet its condition, as to flesh, was hardly changed from birth to death.

The patient was stimulated by external and, as far a practicable, by internal means. Several times, milk was injected into the stomach. Life was for the most part sustained by keeping the body swathed in cloths soaked in cod-liver oil.

An autopsy, ten hours after death, showed the brain and abdominal organs healthy. The lungs were mottled in color, the tints varying in lobules between a light yellow and a light red. There was nowhere the characteristic color of *recent* atelektasis. In the most dependent portions there was a line of *post mortem* engorgement. The anterior third of both lungs was healthy. The posterior two-thirds were more or less collapsed. The surfaces of these portions were irregular; nodulated in lobules—the lobuli were not in the same plane. Some of these were normal; some contained a little air, and others were completely carnified. A moderate inflating force, sufficient to expand the healthy lung of an adult, failed to fill the collapsed portion. Some of the lobuli filled well; others imperfectly; many not at all. The heart was normal—the ductus arteriosus was closed, and the valve of the foramen ovale was perfect, but not adherent in the anterior inferior third of its circumference.

REMARKS.—The original difficulty in the first case was probably indigestion, and consequent diarrhœa and debility: the collapse of the lung following as a consequence of the loss of that physical force necessary for the performance of the respirtory act.

In the second case, from birth there was a lack of vitality. The child could neither swallow nor cough efficiently; its bowels were torpid. This general feebleness invited a collapse of lung tissue, which in turn was a cause of congestion, and of effusion into the respirtory organs—a cumulation of difficulties which could hardly terminate otherwise than in death.

Doubtless many cases of transient cyanosis occurring in infancy, commonly referred to irregularity of the heart's action, are examples of collapse of the lung. The following case, kindly furnished by Dr. GROESBECK, is supposed to be of this character. In a note to the writer, Dr. G. says:—

"The child was naked, the surface cool, skin pale, lips livid; respiration feeble and imperfect, with a mucous rale. While looking at it, the breathing suddenly ceased, the face and lips became more livid, and the child seemed to be dead. In a few moments after, it gasped, and gradually a feeble respiration was established, and the cyanosis disappeared. The physician who arrived before me, finding the throat filled with mucus, had made an effort to dislodge it by means of the finger; tickling the throat with a feather; and turning the child upside down. He then endeavored to inflate the lungs; used the warm bath; and applied hot mustard water to the surface freely.

"This attendant having treated the patient very heroically, I, in accordance with medical usage, adopted an entirely opposite mode of treatment. The child being naked I left it so, but had it wrapped up in warm flannels. Adopting Dr. MEIGS' suggestion in cyanosis, I had the child laid upon the right side, and ordered it to be kept so and not to be disturbed by moving or dressing. I had some food put into a nursing-bottle, and the nipple gently introduced into the child's mouth. It took a teaspoonful, and then had an attack of what I shall call syncope. These faintings occurred frequently during this and the ensuing day. The third day, the child appeared so much better that I had it put to the breast. It nursed vigorously; and from that time recovered. The question now is: What was the cause of this condition? My own opinion is, that it was imperfect expansion of the lungs, ending in collapsed lung and a partial resumption of fatal circulation. The causes that are supposed to favor, or bring about this condition are, among others, speedy birth and exposure to cold, both of which occurred in this case, as the child was suddenly propelled from the brim of the pelvis into the world by one continuous pain. The nurse had not yet arrived; and the child was wrapped in a muslin skirt and laid aside for her to dress. The morning was chilly. The nurse did not arrive until two hours after the birth of the child.

"Here we have two supposed, primal, causal conditions in full action. Dr. JORG says, that in a sudden birth the child does not have the 'besoin de respirer;' and that the fœtal circulation continues in a degree, and consequently the respiration is feeble and insufficient to inflate the lungs; and then we have the train of symptoms which occurred in this case. The depressing effect of cold, enfeebling the respiration, the lungs collapsing gradually from want of air to expand them, would be very likely to establish the fœtal circulation so recently stopped, and the child would exhibit the phenomena stated above."

